# Association between vascular comorbidity and progression of Alzheimer’s disease: a two-year observational study in Norwegian memory clinics

**DOI:** 10.1186/s12877-018-0813-4

**Published:** 2018-05-22

**Authors:** Rannveig Sakshaug Eldholm, Karin Persson, Maria Lage Barca, Anne-Brita Knapskog, Lena Cavallin, Knut Engedal, Geir Selbaek, Eva Skovlund, Ingvild Saltvedt

**Affiliations:** 1 Department of Neuromedicine and Movement Science, Faculty of Medicine and Health Sciences, Norwegian University of Science and Technology (NTNU), 7491 Trondheim, Norway; 2 Department of Geriatrics, St. Olavs Hospital, Trondheim University Hospital, Trondheim, Norway; 3 Norwegian National Advisory Unit on Ageing and Health, Vestfold Hospital Trust, Tønsberg, Norway; 4 Department of Geriatric Medicine, Memory Clinic, Oslo University Hospital, Ullevaal, Oslo, Norway; 5 Department of Clinical Science, Intervention, and Technology, Division of Medical Imaging and Technology, Karolinska Institute, Stockholm, Sweden; 6 Department of Radiology, Karolinska University Hospital, Stockholm, Sweden; 7 Centre for Old Age Psychiatric Research, Innlandet Hospital Trust, Ottestad, Norway; 8 Institute of Health and Society, University of Oslo, Oslo, Norway; 9 Department of Public Health and Nursing, Norwegian University of Science and Technology (NTNU), Trondheim, Norway

**Keywords:** Alzheimer’s disease, Dementia, Mild cognitive impairment, Vascular risk factors, Cardiovascular disease, Cardiovascular risk, Cognitive decline, Progression, Prognosis

## Abstract

**Background:**

Vascular risk factors increase the risk of Alzheimer’s disease (AD), but there is limited evidence on whether comorbid vascular conditions and risk factors have an impact on disease progression. The aim of this study was to examine the association between vascular disease and vascular risk factors and progression of AD.

**Methods:**

In a longitudinal observational study in three Norwegian memory clinics, 282 AD patients (mean age 73.3 years, 54% female) were followed for mean 24 (16–37) months. Vascular risk factors and vascular diseases were registered at baseline, and the vascular burden was estimated by the Framingham Stroke Risk Profile (FSRP). Cerebral medical resonance images (MRIs) were assessed for white matter hyperintensities (WMH), lacunar and cortical infarcts. The associations between vascular comorbidity and progression of dementia as measured by annual change in Clinical Dementia Rating Sum of Boxes (CDR-SB) scores were analysed by multiple regression analyses, adjusted for age and sex.

**Results:**

Hypertension occurred in 83%, hypercholesterolemia in 53%, diabetes in 9%, 41% were overweight, and 10% were smokers. One third had a history of vascular disease; 16% had heart disease and 15% had experienced a cerebrovascular event. MRI showed lacunar infarcts in 16%, WMH with Fazekas score 2 in 26%, and Fazekas score 3 in 33%. Neither the vascular risk factors and diseases, the FSRP score, nor cerebrovascular disease was associated with disease progression in AD.

**Conclusions:**

Although vascular risk factors and vascular diseases were prevalent, no impact on the progression of AD after 2 years was shown.

## Background

Longitudinal studies have established a strong link between vascular risk factors in midlife, such as hypertension, hypercholesterolemia, diabetes mellitus, smoking and being overweight, and development of Alzheimer’s disease (AD) decades later [1]. The mechanisms by which vascular risk factors influence the risk of AD are not entirely understood. Both in vivo and postmortem studies have shown an association between vascular risk factors and amyloid deposition in the brain [2, 3]. Chronic hypertension affects the blood-brain barrier and may alter the clearance of amyloid β (Aβ) [4]. Cerebral blood flow is regulated according to neuronal demands by the neurovascular unit, consisting of microvessels, astrocytes, neurons, and supporting cells that have close anatomical and chemical connections. Reduced cerebral blood flow and dysfunction of the neurovascular unit appear to be key pathways for neuronal damage [5]. In addition, inflammation may be of importance by causing cerebral atrophy and cognitive decline [6, 7]. It is suggested that AD pathology accumulates for many years or even decades before symptoms evolve [8]. However, it is not known if, or to what extent, the presence of vascular risk factors in late life leads to accumulation of AD pathology, as a recent study found no association between late-life vascular risk factors and late-life brain amyloid deposition [2].

As the association between midlife vascular risk factors and incident AD is well established, it could be hypothesised that these risk factors also affect progression after symptoms evolve. A systematic review suggested an association between LDL cholesterol and progression of AD [9], but this has not been a consistent finding in later studies [10–14]. The literature is conflicting regarding which vascular risk factors could be of significance for progression, and even whether these factors seem to ameliorate or aggravate the disease course.

Cerebrovascular diseases are frequent in AD patients, and most elderly AD patients show comorbid brain pathologies in addition to amyloid. In a brain autopsy study with 4629 AD patients, 32% had cerebrovascular disease with a severity that could contribute to their cognitive status while 48% had minor vascular pathology [15]. The threshold at which AD becomes symptomatic is lowered by cerebrovascular disease, and AD pathology is a significant contributor to post-stroke dementia [16, 17]. However, it is not clear whether cerebrovascular disease accelerates disease progression in AD. Although one study showed that white matter intensities (WMH) might exacerbate disease progression in AD dementia [18], most studies on conversion from mild cognitive impairment (MCI) to AD dementia have failed to show that WMH or lacunes are of importance [19].

Extra-cerebral vascular diseases are also associated with cognitive impairment and dementia. Atrial fibrillation (AF) and generalised atherosclerosis increase the risk of cognitive impairment or dementia, and myocardial infarction, peripheral artery disease and congestive heart failure may also be of importance [1, 20]. It may be hypothesised that comorbid cardiovascular disorders increase AD progression through various pathogenetic mechanisms. The consequence of impaired cardiac output may be diminished cerebral blood flow that could increase Aβ generation and reduce clearance of Aβ. Another possible mechanism is neuroinflammation, as chronic systemic inflammation has been shown in AF, heart failure and atherosclerosis [21–23]. Further, an elevated level of inflammation in midlife is associated with smaller brain volume and reduced episodic memory in late life [7]. However, there are few studies on the association between cardiovascular diseases and progression of AD. One study found that AF and angina pectoris were associated with a more rapid decline in cognitive performance, while a history of coronary artery bypass graft surgery was associated with a slower deterioration in AD [24].

Based on the studies mentioned above, it is likely that the total vascular burden, including vascular risk factors and cardiovascular and cerebrovascular disorders, exerts a cumulative effect on the progression of AD. Previous studies have shown an association between composite cardiovascular risk scores in midlife and cognitive decline and incident dementia in late life [25]. However, the relationship between total vascular burden and disease progression in AD patients is less studied [24, 26, 27].

In the Progression of Alzheimer’s Disease and Resource use (PADR) study, we have previously reported considerable heterogeneity in the 2-year progression rate of AD. Increased evidence on predictors of progression in AD is important for individual patients, their families and society, and could even have therapeutic consequences, as these are modifiable and potential targets for intervention. The aim of the study is to investigate whether single vascular risk factors and diseases and total vascular burden are predictors of progression in AD.

## Methods

### Patients

The Progression of Alzheimer’s Disease and Resource Use (PADR) study is a descriptive, longitudinal study conducted in three Norwegian memory clinics, with recruitment of patients between 2009 and 2014. Included were patients with dementia or MCI at baseline, who were home-dwelling, were able to give informed consent and had a proxy who could serve as an informant. Patients who were not fluent in Norwegian, had severe physical illness or lived far away from the memory clinics were excluded.

Patients underwent baseline assessments at the time of diagnostic workup and a follow-up assessment after a mean of 24 months (range 16–37, 80% between 20 and 28 months). The present study included 177 patients with a diagnosis of AD dementia and 105 patients with amnestic MCI (aMCI). The criteria established by the National Institute of Neurological and Communicative Disorders and Stroke-Alzheimer’s Disease and Related Disorders Association (NINCDS-ADRDA) were used for AD dementia [28]. The Winblad criteria were used to diagnose MCI [29]. MCI patients with impaired memory as an early symptom and a score equivalent to or below − 1.5 SD on at least one memory test were categorised as aMCI [30]. The aMCI group was considered to have AD (without dementia). The diagnostic measures of the PADR study and disease progression of the AD patients have been described previously [31].

The study was conducted according to the Helsinki declaration, participation was voluntary, and patients received oral and written information and gave written consent to participate. Only patients with capacity to consent at baseline were recruited. The Regional Committee for Medical and Health Research Ethics in South East Norway approved the study.

### Assessments

Patients underwent comprehensive neuropsychological and physical examinations at baseline, according to a standardised research manual, with most of the tests repeated at follow-up. The evaluation also included measurements of height, weight, systolic and diastolic blood pressure, and in most cases an electrocardiogram (ECG). Demographic data, medical history, smoking status and drugs in current use were recorded. The cognitive test battery included the Mini-Mental State Examination (MMSE) (0–30) [32]. The primary outcome measure was the Clinical Dementia Rating scale Sum of Boxes (CDR-SB). The CDR evaluates six areas of cognition and function and assesses the severity of impairment on the range from normal cognitive function to severe dementia. Each of six items is scored 0–3 and item scores can be summed to produce a continuous scale, CDR-SB (0–18, higher scores denoting more severe impairment) [33].

Blood sample analyses included cholesterol, creatinine and glucose. Estimated glomerular filtration rate (eGFR) was calculated based on serum or plasma creatinine using the Chronic Kidney Disease Epidemiology Collaboration (CKD-EPI) equation. Apolipoprotein E (ApoE) genotyping was conducted using the Illumina Infinium OmniExpress v1.1 chip at deCODE Genetics, Reykjavik, Iceland, and the result was dichotomised based on the presence of at least one ApoE epsilon 4 (ApoE ε4) allele into carriers and non-carriers of ApoE ε4. Structural brain imaging was performed for all patients at baseline, using magnetic resonance imaging (MRI) in most cases (87%); however, some patients underwent computed tomography (CT) scans because of pacemakers or claustrophobia.

### Vascular risk factors

Patients were considered as having hypertension when reported in their medical history, if using antihypertensives (angiotensin-converting enzyme inhibitors, angiotensin II receptor antagonists, beta blockers, diuretics or calcium antagonists), or if they had a blood pressure of > 140 systolic or > 90 diastolic at baseline. Patients had hypercholesterolemia if this was reported in the medical history, they used statins or had a total cholesterol level of ≥6.5 mmol/l at baseline. Diabetes was registered by medical history or the use of any antidiabetic drug. Being overweight was defined as having a body mass index ≥25 kg/m^2^, and patients were registered as smokers if they smoked at baseline, regardless of former smoking history. AF was recorded from the medical history and ECGs. Information on previous strokes, transient ischemic attacks, heart disease (angina pectoris, myocardial infarction, heart failure or valvular disease), and peripheral artery disease was retrieved from the hospitals’ medical records.

To assess the vascular burden of each patient, the Framingham Stroke Risk Profile (FSRP) was calculated. The score integrates the effect of age, sex, and measurements of systolic blood pressure, the use of antihypertensive treatment, diabetes mellitus, current smoking status, prevalent cardiovascular disease, current or previous AF, and the presence or absence of left ventricular hypertrophy on ECG. Cardiovascular disease is defined in this risk score as a history of myocardial infarction, angina pectoris, intermittent claudication or congestive heart failure. ECGs were examined for left ventricular hypertrophy according to the Framingham criteria [34]. In addition to the original FSRP, risk scores for all patients were calculated with the recently published revised FSRP, based on more current data on stroke risk factors, with a lower impact of AF and prevalent cardiovascular disease [35].

Blood pressure measurements were missing for three, smoking status for seven, weight and height for 26, creatinine for ten, cholesterol measurements for 48, and an ECG for 71 patients. When measurements were not available, vascular risk factor status was based on medical history and drug use alone.

### MRI scans

An experienced neuroradiologist examined the MRI scans blinded to all clinical data. Medial temporal atrophy (MTA) was rated using the Scheltens MTA scale [36], which includes evaluation of the choroid fissure, the temporal horn of the lateral ventricle, and the height of the hippocampus, yielding a score of 0–4 (higher score denoting more atrophy). MTA was assessed on the left and right sides separately, and the mean score was calculated. WMHs were evaluated using the Fazekas scale, rating severity of WMHs in the periventricular and subcortical regions combined on a scale of 0–3 [37]. The presence and number of lacunes (≤10 mm) and cortical infarcts were recorded.

The MRI examinations were regular clinical examinations conducted at several different centres using different MRI protocols. Only MRIs performed within 6 months before or after the clinical assessment at baseline were included for analyses (mean 2.3 months (SD 1.5), within < 4 months in 88% of participants), resulting in MRIs from 206 AD patients.

### Statistics

Descriptive statistical methods were used for baseline characteristics. For comparisons between groups, independent samples t-tests were used for continuous data, and Pearson’s χ^2^ test was used for categorical data. Change from baseline to follow-up was calculated by dividing CDR-SB score differences by time in years. Follow-up time was checked for normal distribution.

Linear regression analyses were conducted with the progression as measured by annual CDR-SB change as the dependent variable. First, unadjusted analyses were carried out for vascular risk factors, vascular diseases, FSRP and MRI findings. Interactions between vascular factors and ApoE ε4 carrier status and disease stage (MCI or dementia) were explored and were not significant. All analyses except FSRP were then adjusted for age and sex. Subgroup analyses were performed on patients found to have hypertension, hypercholesterolemia or diabetes that was not treated at baseline, in order to investigate the potential effect of untreated risk factors on the progression of AD.

Data were analysed using IBM SPSS Statistics for Windows, version 24.0, Armonk, NY, USA. Statistical significance was defined as *p* < 0.05.

## Results

The mean age of the study patients was 73.3 (SD 8.8) years, 54% were women, 61% were apoE ε4 carriers, and the mean MMSE score was 23.7 (SD 4.4) (Table 1). In the study sample, 83% had hypertension, 53% hypercholesterolemia, 9% diabetes, 41% were overweight and 10% smokers. Only 6 % had none of these vascular risk factors, whereas 24% had one, 45% two, 20% three, 5 % had four, and none had all five of them. A history of vascular disease was present in 33% of the patients, the most common being heart disease (16%) and cerebrovascular events (15%). On MRI, 16% had lacunar and 4 % had cortical infarcts. WMHs with a Fazekas score of 2 were present in 26, and 33% had WMHs with a Fazekas score of 3.

**Table 1:** Clinical and demographic characteristics at baseline

	Total		
Age, years, mean (SD)	282	73.3	(8.8)
Female, n (%)	282	153	(54.3)
Education, years, mean (SD)	282	11.7	(3.6)
Drugs in regular use, mean (SD)	280	3.1	(2.6)
Antihypertensives, number of users (%)	280	137	(48.9)
Statins, number of users (%)	280	85	(14.7)
Antidiabetic agents (number of users, %)	280	20	(7.1)
Antithrombotic agents (number of users, %)	280	131	(46.8)
Platelet inhibitors (number of users, %)	280	114	(40.4)
Anticoagulants (number of users, %)	280	21	(7.5)
ApoE ε4 carrier, n (%)	252	154	(61.1)
MMSE (0–30), mean (SD)	280	23.7	(4.4)
CDR sum of boxes (0–18), mean (SD)	282	4.2	(2.8)
Vascular risk factors (VRFs)			
Hypertension, n (%)	282	234	(83.0)
Hypercholesterolemia, n (%)	282	149	(52.8)
Diabetes mellitus, n (%)	282	26	(9.2)
Overweight (BMI > 25), n (%)	256	106	(41.4)
Smoking (current), n (%)	275	26	(9.5)
Framingham Stroke Risk Profile^b^ (0–1), women, mean (SD)	113	0.21	(0.14)
Framingham Stroke Risk Profile^b^ (0–1), men, mean (SD)	92	0.26	(0.16)
Vascular diseases			
Atrial fibrillation, n (%)	282	28	(9.9)
History of cerebrovascular event (stroke or TIA), n (%)	282	43	(15.2)
History of heart disease, n (%)	282	46	(16.3)
Left ventricular hypertrophy in ECG^a^, n (%)	211	8	(3.8)
History of peripheral vascular disease, n (%)	273	7	(2.6)
Estimated GFR < 60 ml/min/1.73m^2^, n (%)	273	47	(17.2)
MRI findings			
Medial temporal lobe atrophy (mean of both sides), mean (SD)	203	1.9	(0.9)
Cortical infarcts, n (%)	206	9	(4.4)
Lacunar infarcts, n (%)	206	33	(16.0)
White matter hyperintensities			
Fazekas 0 or 1, n (%)	205	84	(40.9)
Fazekas 2, n (%)	205	53	(25.9)
Fazekas 3, n (%)	205	68	(33.2)
Cerebrovascular disease: cortical infarcts, lacunes or Fazekas ≥2, n	206	128	(62.1)
Cerebrovascular disease: cortical infarcts, lacunes or Fazekas 3, n	206	88	(42.7)

^a^Framingham criteria

^b^Ten year probability of stroke

Unadjusted regression analyses showed no significant associations between any individual vascular risk factor and progression of AD (not shown). There was a trend for patients with high BMI to progress less than others (*p* = 0.09), and the same trend was found for patients with three or more vascular risk factors (*p* = 0.16). Results adjusted for age and sex are shown in Table 2. There was no significant association between the FSRP score and progression of AD (Table 2). The revised FSRP score gave significantly lower stroke risk estimates for both men and women and did not show any significant association with AD progression (not shown). The existence of cerebrovascular disease on MRI, as infarcts (either cortical or lacunar) or WMHs, was not found to predict progression of AD.

**Table 2:** Associations between vascular risk factors at baseline and progression measured by annual change in CDR-SB

	Age and sex-adjusted			
	Beta	95% CI	p	R^2^
Vascular risk factors (VRFs)				
Hypertension	−0.049	(−0.617, 0.519)	0.87	0.04
Hypercholesterolemia	0.262	(−0.153, 0.676)	0.22	0.05
Diabetes mellitus	0.128	(−0.592, 0.848)	0.73	0.04
Overweight (BMI ≥25)	− 0.391	(− 0.840, 0.059)	0.09	0.04
Smoking (current)	0.050	(−0.677, 0.778)	0.89	0.04
≥ 2 VRFs	0.121	(−0.370, 0.612)	0.63	0.03
≥ 3 VRFs	−0.371	(−0.889, 0.147)	0.16	0.04
Framingham Stroke Risk Profile, women*	−0.297	(−2.769, 2.175)	0.81	0.00
Framingham Stroke Risk Profile, men*	0.930	(−1.638, 3498)	0.47	0.01
ApoE ε4 carrier	0.384	(−0.069, 0.836)	0.10	0.06
Vascular diseases				
Heart disease	0.196	(−0.380, 0.771)	0.50	0.04
Atrial fibrillation	−0.159	(0.902, 0.583)	0.67	0.04
Peripheral vascular disease	0.512	(−0.834, 1.858)	0.46	0.04
Cerebrovascular event (stroke and/or TIA)	−0.315	(−0.896, 0.265)	0.29	0.04
MRI findings				
Cortical infarcts (1 = cortical infarcts, 0 = no cortical infarcts)	0.707	(−0.518, 1.932)	0.26	0.04
Lacunes (1 = ≥1 lacune on MRI, 0 = no lacunes)	−0.177	(−0.867, 0.514)	0.61	0.04
Infarcts (cortical and/or lacunes)	0.038	(−0.611, 0.687)	0.91	0.03
White matter hyperintensities Fazekas 3	−0.291	(−0.872, 0.290)	0.32	0.04
White matter hyperintensities Fazekas 2 or 3	−0.207	(−0.776, 0.362)	0.47	0.04
Infarcts and/or WMH Fazekas 3	−0.079	(−0.625, 0.468)	0.78	0.03
Infarcts and/or WMH Fazekas 2 or 3	−0.298	(−0.872, 0.277)	0.31	0.04
MTA (mean of right and left side)	0.036	(−0.262, 0.334)	0.81	0.04

* The Framingham Stroke Risk Profiles were not adjusted for age and sex

Regression analyses were additionally performed on subgroups of patients with untreated hypertension, hypercholesterolemia or diabetes, but no association with the progression of AD was found (not shown).

## Discussion

In this descriptive cohort study in memory clinic patients with AD, we examined whether vascular risk factors, cardiovascular and cerebrovascular diseases or total vascular burden were of importance for the progression of AD. None of these was shown to predict progression of AD. Prevalence of vascular risk factors and vascular diseases was high in the study population, and many patients received treatment for these disorders at baseline.

Given the strong link between vascular risk factors in midlife and later development of AD, the lack of association between late-life vascular burden and disease progression in AD is surprising. Although single risk factors might not have sufficient power to determine AD progression, we would have expected a combination of risk factors or diseases to show an effect. However, as no consistent effect of any vascular risk factor has been identified across studies, it is possible that vascular risk factors do not influence the progression of AD in the symptomatic stage [10, 14]. The time lag between midlife exposure to vascular risk factors and development of AD may be several decades. If the effect of vascular risk factors is mediated through slowly evolving processes, as amyloid accumulation and development of WMHs, the time may be too short to observe a difference from vascular risk factors in late life [2]. An alternative explanation may be that when the amount of AD pathology surpasses a certain threshold, as may be the case for many patients with AD dementia, the influence of AD pathology may be so strong that an additional effect of vascular disease cannot be discerned [38, 39].

Contrary to our hypothesis, we observed a trend for slower disease progression in the patients with the highest number of vascular risk factors. It is possible that this group will have more vascular pathology, contributing to the initial diagnosis and severity of cognitive impairment or dementia, but to a lower burden of AD pathology. This may explain an apparent protective effect of vascular risk factors on cognitive decline. Alternatively, successful treatment of vascular risk factors may have influenced the disease course.

Although obesity is considered a cardiovascular risk factor, in the present study, we found a trend for patients with high BMI to progress less than patients of normal weight. This has been observed in other studies and may be a result of reverse causality [40].

Despite the high prevalence of cerebrovascular disease among study patients, we were not able to demonstrate any association between MRI findings of cerebrovascular disease and progression of AD. As we know that the coexistence of cerebrovascular disease lowers the symptomatic threshold of AD [17], our hypothesis was that these patients would experience a more rapid progression. However, this may not be the case, as they may have developed cognitive impairment with less underlying AD pathology than others. Longitudinal studies indicate that the reduction in cognitive function associated with cerebrovascular injury remains relatively stable over time in old age, whereas the AD pathology leads to increasing impairment [41].

As only half of the study patients showed more than minimal progression over 2 years [31], our study may have lacked sufficient power to show an effect of vascular burden. If the impact of vascular burden emerges very gradually, e.g. as a result of amyloid accumulating slowly, the follow-up time of 2 years may be too short. We did not find an association between untreated risk factors and progression of cognitive decline. However, many of our patients received treatment for their vascular risk factors at baseline, and the negative findings may be a consequence of optimal treatment.

Another explanation for the lack of associations in the present study may be related to characteristics of the study population, who were memory clinic patients, younger, more educated and with milder AD than the general AD population. We have previously shown that the overall progression rate of AD patients in the PADR study was similar to what has been reported in other studies [31]. The prevalence of vascular risk factors and diseases increases with age, as does the proportion of AD patients who have comorbid vascular brain pathology. Seven percent of the study patients died before follow-up; some of them may have died of vascular disease.

Vascular risk factors and diseases were frequent in the study sample and comparable to other studies [11, 13]. In comparison with elderly participants in a Norwegian population study [42], the prevalence of diabetes and hypercholesterolemia was similar, while hypertension, coronary artery disease and strokes were more common among our study patients. On the other hand, fewer of our patients were overweight or smoked. Despite the high prevalence of hypertension in our study, there were few cases of left ventricular hypertrophy and low numbers of chronic kidney disease (Table 2), indicating successful antihypertensive treatment. The frequency of ApoE ε4 carriers was similar to other studies in AD patients [43]. Thus, we found no differences in vascular burden between our study and other AD studies that could explain the lack of association between vascular burden and progression of AD among our patients.

It can be questioned whether the FSRP represents a good proxy measure of vascular burden for our study. The FSRP evaluates only the current levels of risk factors and does not take into account previous risk levels or vascular disease in the brain. Because several vascular risk factors tend to decrease in the years leading up to dementia [44], the FSRP score may not give optimal risk estimates when used in the memory clinic.

The inclusion criteria and the attrition rate limit the generalisability of the study. Although patients lost to follow-up did not differ in comorbidities compared to the patients who remained in the study, the prevalence of smokers was twice as high in this group. We may have missed unrecognised type 2 diabetes as a risk factor, as we did not screen for this. The MRI for the study was performed as clinical procedures, not standardised for research, and was not repeated at follow-up. Thus, analyses on progression in relation to change in MRI findings could not be conducted. Although we recorded the medical history of all patients, information regarding duration and timing of risk factors was seldom available.

## Conclusion

The present study found no association between vascular risk factors and cerebrovascular disease and progression of AD. Further clinical studies with larger sample sizes and longer follow-up are needed to clarify whether vascular conditions may influence disease progression in AD. The clinical question remains if and how we should treat AD patients with vascular risk factors to prevent disease progression, and future studies are needed to answer this.

## Abbreviations

AD: Alzheimer’s disease
ApoE ε4: Apolipoprotein E epsilon 4
ApoE: Apolipoprotein E
CKD-EPI: Chronic Kidney Disease Epidemiology Collaboration
CDR: Clinical Dementia Rating Scale
CDR-SB: Clinical Dementia Rating scale Sum of Boxes
CT: Computer tomography
ECG: Electrocardiogram
FSRP: Framingham Stroke Risk Profile
ICD-10: International Classification of Diseases 10th revision
MCI: Mild cognitive impairment
MMSE: Mini-Mental State Examination
MRI: Magnetic resonance imaging
NINCDS-ADRDA: National Institute of Neurological Disorders and Stroke-Alzheimer Disease and Related Disorders
PADR: Progression of Alzheimer’s Disease and Resource use
REC: Regional Committees for Medical and Health Research Ethics
SD: Standard deviation
VRF: Vascular risk factor
WMH: White matter hyperintensities
